# Quality of Life after Stapled Hemorrhoidopexy: A Prospective Observational Study

**DOI:** 10.1155/2013/903271

**Published:** 2013-08-22

**Authors:** Pankaj Kumar Garg, Gopal Kumar, Bhupendra Kumar Jain, Debajyoti Mohanty

**Affiliations:** Department of Surgery, University College of Medical Sciences and Guru Teg Bahadur Hospital, University of Delhi, DIlshad Garden, Delhi 110095, India

## Abstract

*Objective*. The objective of the study was to assess the change in quality of life (QOL) of patients undergoing stapled hemorrhoidopexy (SH) using WHO Quality of Life-BREF (WHOQOL-BREF) questionnaire. *Methods*. The study sample comprised patients with symptomatic II, III, and IV degree hemorrhoids, undergoing SH. The patients were asked to complete WHOQOL-BREF questionnaire before and one month following the surgery. *Result*. There were 20 patients in the study group. The postoperative pain score measured by visual analogue scale at six hours postoperatively was 7.60 ± 1.23, which reduced to 0.70 ± 0.92 at 24 hours. The items in the WHOQOL-BREF had high-internal consistency or reliability as shown by high Cronbach's alpha coefficient which was 0.82 and 0.90 for pre- and postoperative questionnaires. There was significant improvement in the overall perception of QOL and health, and in physical and psychological domains. There was modest improvement in environmental domain, while no change was noted in social domain. *Conclusion*. SH improved the quality of life of patients treated for hemorrhoids.

## 1. Introduction

Hemorrhoids are one of the common conditions encountered in general surgical clinics. Large numbers of patients who have hemorrhoids are asymptomatic. Bleeding during defecation is the most frequent presenting symptom. The hemorrhoids may prolapse (grade II–IV) and result in other symptoms of mucus seepage, pruritis, loss of discrimination and continence to flatus, and occasional fecal incontinence. The latter may cause social embarrassment. Patients with permanent prolapsed hemorrhoids may also face difficulty in maintaining local hygiene. Pain is not a usual accompaniment of hemorrhoids but thrombosed hemorrhoids become painful. These symptoms impact the quality of life of patients. The need for surgical treatment is based on the effect of the symptoms on the quality of life of the patient [1]. Currently, several operative procedures are in vogue: excisional hemorrhoidectomy, stapled hemorrhoidopexy, and selective hemorrhoidal artery ligation. The stapled hemorrhoidopexy scores over excisional hemorrhoidectomy in terms of less postoperative pain, absence of perineal wound requiring care extending over a couple of weeks, and early return to normal day-to-day routine. The present study was conducted to assess the quality of life of the patients after stapled hemorrhoidopexy (SH) for hemorrhoids, with the help of WHO Quality of Life-BREF questionnaire.

## 2. Methods

This prospective observational study was conducted in the Department of Surgery of a tertiary teaching hospital in north India. All the patients with symptomatic II, III, and IV degree hemorrhoids undergoing stapled hemorrhoidopexy were studied. In order to achieve a minimum 50% change in quality of life after SH, a sample size of 259 was required to have 5% level of significance (two sided), power of 90%, and confidence interval of 40–60% [2]. As this was a time-bound project (two-month ICMR-STS project), 20 patients were included in the study. The inclusion criteria were: age of 18–65 years, symptomatic II, III, and IV degree hemorrhoids, and fitness for anaesthesia under ASA grade I and II. The exclusion criteria were: thrombosed hemorrhoids, concomitant perianal fistula, fissures, or abscesses; proposed second procedure under same anesthesia, and unwillingness to give consent to participate in the study.

The clearance for the study was obtained from the Institutional Ethical Committee-Human Research. An informed written consent was taken from the patients after explaining the procedure, expected outcome, and possible side effects in detail. Procedure was performed under general or regional anesthesia. The SH procedure was performed according to Longo's technique [3]. The patients were administered an injection of diclofenac sodium (1.5 mg/Kg) at the end of the surgery. Thereafter, they were advised analgesics on demand. Patient's demographic data, operative time, and postoperative complications including pain intensity on VAS, bleeding, urinary retention, painful defecation, and duration of hospital stay were recorded. The patients were discharged when they felt comfortable and pain intensity on VAS was <3. The patients were called weekly for one month in the follow-up clinic.

Quality of life (QOL) assessment was done by WHO Quality of Life-BREF (WHOQOL-BREF) questionnaire [4]. The Hindi version of the WHOQOL-BREF questionnaire was used for evaluating the QOL. The validity and reliability of the Hindi version of the WHOQOL-BREF have already been tested [5]. It consists of 24 items, covering four domains: physical condition, psychological condition, social relationships, and environmental issues. The physical domain has questions related to daily activities, treatment compliance, pain and discomfort, sleep and rest, energy, and fatigue. The psychological domain assesses positive and negative feelings, self-esteem, body image and physical appearance, personal beliefs, and attention. The social relationship domain covers personal relationships, social support, and sexual activity. The environmental domain explores physical security, financial resources, health and social care and their availability, opportunities for acquiring new information and skills, and opportunities for and participation in recreation and transport. Besides these domains, two additional questions were used: “How would you rate your QOL?” and “How satisfied are you with your health?” The final questionnaire, thus, contained 26 items. Each item used a 5-point Likert scale. For example: 5 = very satisfied, 4 = satisfied, 3 = neither satisfied nor dissatisfied, 2 = dissatisfied, and 1 = very dissatisfied. The high scores indicate a better QOL. For comparing the domain scores before and after SH, the WHOQOL-BREF scores were converted into scores from 0 to 100, with a lowest score of zero and a highest score of 100. The patients were asked to complete WHOQOL-BREF questionnaire before and one month after the surgery.

*Statistical Analysis*. Data were managed on an Excel spreadsheet. Descriptive analysis of demographic data, clinical parameters, postoperative complications, and WHOQOL-BREF questionnaire scores was carried out. Quantitative variables were summarized by mean and standard deviation or median and interquartile range, and categorical variables were summarized by frequency (percentage). Wilcoxon signed rank test was used for comparing scores of various domains of WHOQOL-BREF questionnaire. *P* values less than 0.05 were considered as statistically significant. Statistical analysis was done using SPSS16 software.

## 3. Results

The mean age of the patients was 44.30 ± 13.71 years, and male-to-female ratio was 14 : 6. The median duration of symptoms was 21 (interquartile range 6–30) months. Prolapse of the hemorrhoidal mass was reported by 14 patients. All the operations were completed uneventfully. The mean duration of surgery was 27.50 ± 3.84 minutes. Doughnut was complete in all the patients.  Table 1 shows the postoperative pain score at VAS at different points of time. The VAS score at six hours postoperatively was 7.60 ± 1.23, which reduced to 0.70 ± 0.92 at 24 hours. None of the patients had VAS score of ≤3 at six hours postoperatively; however, all 20 patients were reported to have VAS score of ≤3 at 24 hours postoperatively. In immediate postoperative period, two patients had urinary retention and required catheterization. There was minimal blood staining of the dressing of eight patients. Fifteen patients had bowel motion within 24-hour postoperative hospital stay; four of them complained of painful defecation. All the patients could be discharged from the hospital on the first postoperative day.  Table 2 shows the postoperative complications as noticed by the patients during weekly postoperative follow-up.

## 4. Quality of Life Score

The items in the WHOQOL-BREF had high-internal consistency or reliability as shown by high Cronbach's alpha coefficient which was 0.82 and 0.90 for pre- and postoperative questionnaire.  Table 3 shows the change in QOL scores in various domains. There was significant improvement in overall perception of QOL and health and in physical and psychological domains. There was modest improvement in environmental domain, while no improvement was seen in social domain.

## 5. Discussion

Hemorrhoids are equally distributed in men and women, and the incidence increases with age. Our study group consisted of middle-aged individuals with men outnumbering women. The possible explanation may be that women, in the conservative Indian society, are less forthcoming with the problems involving the perineal area. The patients in our study presented with a long median duration of 21 months. Our hospital caters mostly to people of lower socio-economic stratum who work on daily wage basis to earn their livelihood. These people often choose to ignore the intermittent episodes of painless bleeding per rectum to avoid loss of wage on the day of hospital visit. They seek medical advice only when the symptoms become exaggerated.

Several studies have been undertaken to compare the SH with excision hemorrhoidectomy in terms of operating time, hospital stay, postoperative pain, anal discharge, postoperative bleeding, difficulty in defecation, stenosis, incontinence, and so forth [6]. We have evaluated SH through a different lens: the quality of life which is being increasingly visualized as a more refined and realistic parameter of success of any medical intervention. Hemorrhoids may adversely affect the quality of life to varying extend depending on the degree of hemorrhoids and symptoms associated with them. 

Overall perception of QOL and health and the physical, psychological, social, and environmental domain are affected to different extent as is evident from the preoperative assessment of QOL in our patients (Table 3). 

The change in the QOL score after SH is a reflection of two distinct elements: (a) alleviation of symptoms of hemorrhoids following surgery and (b) postoperative pain and sequel/complications following SH. No patient reported anal bleeding after 2 weeks. The postoperative pain rapidly decreases in severity to the VAS score of 2 or less at 24 hours following SH. This has facilitated early discharge of all the patients on the first postoperative day. The occurrence of other postoperative complications is shown in Table 2. At 4 weeks after surgery, only one of the twenty patients reported a complaint (constipation). The QOL score, at one month after SH, showed improvement as compared to preoperative score in respect of five of the six parameters of WHOQOL-BREF Questionnaire (Table 3). However, no change was found in respect of social domain.

The WHO defines QOL as “individuals' perceptions of their position in life in the context of the culture and value systems in which they live, and in relation to their goals, expectations, standards and concerns” [7]. The purpose is to ensure satisfaction with the everyday life as a whole. The QOL scores assume importance in the present day scenario due to the changing pattern of health care delivery. The focus of health care is on the patient as a whole rather than to simply eliminate the disease process. We compared the various WHOQOL-BREF parameters before and one month after SH. The major factors responsible for significant improvement in overall perception and physical and psychological domains were: relief of the symptoms of hemorrhoids and the minimal postoperative pain. This allowed the patients greater freedom to go through their daily routine and ability to focus better on their work. We are unable to find any Indian study addressing this important issue; however, a European study of QOL (using QLQ-C30 form) following SH recorded a decrease in the overall QOL on the day of surgery. The score returned to the preoperative level on the 7th postoperative day and reached a significantly higher level at the end of one month [8]. Low postoperative pain intensity and freedom from wound care were cited as the reasons behind this improved outcome. A long follow-up period is suggested by some authors to observe the sustained improvement in the QOL parameters in patients undergoing SH [8, 9]. Our patients reported no change in their social domain. The social domain covered personal relationship, sex life, and supports from friends. Concern to protect the operated region could restrict sexual activities. Proper counseling of the patients preoperatively and at the time of discharge could make the patients more comfortable in the perioperative period and improve the quality of life. A powerful prospective study with a larger sample size is the need of the hour to address the various QOL issues faced by patients in this part of the world.

## Figures and Tables

**Table 1:** Postoperative pain intensity on VAS score.

Time point (in hours)	VAS score (median ± IQR)	Patients with VAS score of ≤3
6	8 (7-8)	0/20
12	5 (4–7)	4/20
18	2 (0–5)	12/20
24	0 (0–2)	20/20

**Table 2:** Postoperative complaints as recorded by the patients at weekly follow-up.

Complication	Postoperative period (in weeks)			
	1	2	3	4
Bleeding	4/20	1/20	0/20	0/20
Constipation	6/20	4/20	2/20	1/20
Urgency	6/20	0/20	0/20	0/20
Painful defecation	4/20	0/20	0/20	0/20

**Table 3:** Changes in various domains of QOL.

	Preoperative		Postoperative		Wilcoxon signed rank test, *P* value
	Median score	IQR	Median score	IQR	
Overall perception of QOL	75.0	50.0–75.0	100.0	75.0–100.0	**0.00**
Overall perception of health	75.0	50.0–75.0	100.0	56.2–100.0	**0.03**
Physical domain	57.1	46.4–71.4	76.7	67.8–89.2	**0.00**
Psychological domain	75.0	66.7–83.3	79.1	70.8–91.6	**0.00**
Social domain	75.0	66.7–83.3	75.0	66.7–71.87	0.15
Environmental domain	62.5	53.1–71.8	67.1	62.5–71.8	**0.04**
